# Intraventricular cerebrospinal fluid temperature analysis using MR diffusion-weighted imaging thermometry in Parkinson's disease patients, multiple system atrophy patients, and healthy subjects

**DOI:** 10.1002/brb3.340

**Published:** 2015-04-24

**Authors:** Kaoru Sumida, Noriko Sato, Miho Ota, Koji Sakai, Yasumasa Nippashi, Daichi Sone, Kota Yokoyama, Kimiteru Ito, Norihide Maikusa, Etsuko Imabayashi, Hiroshi Matsuda, Kei Yamada, Miho Murata, Akira Kunimatsu, Kuni Ohtomo

**Affiliations:** 1 Department of Radiology, National Center Hospital of Neurology and Psychiatry4-1-1 Ogawa-Higashi-Cho, Kodaira, Tokyo, 187-8502, Japan; 2 Department of Radiology, Graduate School of Medicine and Faculty of Medicine, The University of Tokyo7-3-1 Hongo, Bunkyo-ku, Tokyo, 113-8655, Japan; 3 Department of Mental Disorder Research, National Institute of Mental Health4-1-1 Ogawa-Higashi-Cho, Kodaira, Tokyo, 187-8502, Japan; 4 Department of Human Health Science, Graduate School of Medicine, Kyoto University53 Shogoin Kawahara-cho, Sakyo-ku, Kyoto, 606-8507, Japan; 5 Department of Psychiatry, National Center Hospital of Neurology and Psychiatry4-1-1 Ogawa-Higashi-Cho, Kodaira, Tokyo, 187-8502, Japan; 6 Department of Radiology, Tokyo Metropolitan Geriatric Hospital35-2 Sakae-Cho, Itabashi-ku, Tokyo, 173-0015, Japan; 7 Integrative Brain Imaging Center, National Center of Neurology and Psychiatry4-1-1 Ogawa-Higashi-Cho, Kodaira, Tokyo, 187-8551, Japan; 8 Department of Radiology, Graduate School of Medical Science, Kyoto Prefectural University of Medicine465 Kajii-cho, Kawaramachi-honmachi, Kamigyo-ku, Kyoto, 602-8566, Japan; 9 Department of Neurology, National Center Hospital of Neurology and Psychiatry4-1-1 Ogawa-Higashi-Cho, Kodaira, Tokyo, 187-8502, Japan

**Keywords:** Cerebrospinal fluid, diffusion-weighted imaging thermometry, multiple system atrophy, Parkinson's disease, temperature

## Abstract

**Purpose:**

We examined the temperature of the intraventricular cerebrospinal fluid (T_v_) in patients with Parkinson's disease (PD) and those with multiple system atrophy (MSA) in comparison with healthy subjects, and we examined normal changes in this temperature with aging.

**Methods:**

T_v_ was estimated by magnetic resonance (MR) diffusion-weighted imaging (DWI) thermometry in 36 PD patients (19 males, 17 females), 34 MSA patients (17 males, 17 females), 64 age-matched controls (27 men, 37 women), and 114 all-age adult controls (47 men, 67 women; 28–89 years old). The volume of lateral ventricles was also estimated using FreeSurfer in all subjects. T_v_ and ventricular volume data were compared among the PD and MSA patients and age-matched controls. We also evaluated the relationship between T_v_ and age in the 114 all-age controls, controlling for ventricular volume. Men and women were analyzed separately.

**Results:**

The male PD and MSA patients had significantly higher T_v_ values compared to the male controls, with no significant difference in ventricular volume among them. There was no significant difference in T_v_ between the female patients and controls. In the all-age male controls, there was a significant negative correlation between T_v_ and age controlling for ventricular volume, and this was not observed in the women.

**Conclusion:**

DWI thermometry is a useful and easy method for demonstrating an altered intracranial environment in male patients and healthy controls, but not in females. DWI thermometry can thus be used to help to explore the pathophysiology of Parkinsonian syndromes and to differentiate individuals affected by neurodegenerative disease with autonomic dysfunction from those without it.

## Introduction

Parkinson's disease (PD) is a progressive neurodegenerative movement disorder characterized by rigidity, tremor, and bradykinesia. Its prevalence increases with age, and it affects 1% of the population over age 65 (Aarsland et al. 2001). Multiple system atrophy (MSA) is one of the diseases that present as a Parkinsonian syndrome. It is an adult-onset, sporadic, progressive neurodegenerative disease characterized by Parkinsonian features of varying severity, including cerebellar ataxia, autonomic failure, and corticospinal disorders. Depending on the predominant symptoms, MSA is divided into the Parkinsonian type (MSA-P) and the cerebellar type (MSA-C) (Quinn 1986; Wenning et al. 1997; Geser et al. 2006; Gilman et al. 2008). One important PD-related disorder is autonomic failure caused by postganglionic denervation. Autonomic failure is also present in MSA, although it is mainly due to preganglionic failure (Iodice et al. 2011; Miyoshi et al. 2013; Sakakibara et al. 2014).

The brain is one of the most energy-demanding organs, and most of the energy used for cerebral metabolism is eventually released as heat. As the brain is a very heat-sensitive organ, the maintenance of a stable temperature is crucial for it to function normally. In the central autonomic network, the hypothalamic-brainstem-spinal-cord loop plays a role in thermoregulation, controlled through the activity of neurons at the preoptic-anterior hypothalamus, known as the nucleus preopticus periventricularis (NPP), which determines the body temperature set-point (Dougherty 1999). PD and MSA are neurodegenerative diseases that interfere with the central autonomic system (Cohen et al. 1987; Magalhaes et al. 1995). The question thus arises as to whether cerebral temperature changes occur in PD and MSA.

The temperature of the intraventricular cerebrospinal fluid (T_v_) has been estimated easily and noninvasively using magnetic resonance (MR) diffusion-weighted imaging (DWI) (Yamada et al. 2010; Sakai et al. 2011, 2012; Ota et al. 2014; Sai et al. 2014; Tazoe et al. 2014). Although the regional cerebral temperature can also be directly measured using magnetic resonance spectroscopy (MRS), much more time is needed to perform an MRS examination compared to MR-DWI. DWI is already a routine sequence that is easily performed as part of a clinical examination and its findings can be reviewed retrospectively. DWI thermometry thus has the potential to be a clinically useful method. However, it measures the cerebrospinal fluid (CSF) in the ventricles (Nagy et al. 2009; Kozak et al. 2010; Sakai et al. 2012), so it may be more influenced by body core temperature and CSF circulation than MRS is. Therefore, when interpreting DWI thermometry, these aspects must be kept in mind. For the elucidation of the significance of DWI thermometry, it is necessary to determine the behaviors of T_v_ in various situations.

The aim of this study was to assess the T_v_ of PD and MSA patients in comparison with normal controls using DWI thermometry. We also evaluated the changes of T_v_ in normal adult controls including those over the age of 60, considering ventricular size.

## Materials and Methods

### Patients and controls

MR images of patients with PD or MSA were taken for a retrospective analysis. A review of our radiological reporting system revealed the cases 120 suspected-PD and 53 suspected-MSA patients who underwent MRI on a 3T system between September 2012 and June 2014 at a single institution. Among them, 62 PD and 45 MSA patients were clinically confirmed. The clinical diagnoses were made by expert neurologists, and standard diagnoses of PD and MSA were made according to the United Kingdom Parkinson's Disease Society Brain Bank Clinical Diagnostic Criteria (steps one and two) (Hughes et al. 1992) and the second consensus clinical criteria for MSA (Gilman et al. 2008), respectively. Exclusion criteria were neurological disorders other than PD or MSA; Mini-Mental State Examination score <24 or a diagnosis of dementia; febrile state or medical condition known to affect body temperature (e.g., infection, cancer); and premenopausal women. Expert radiologists confirmed the MR imaging features that support MSA (i.e., linear hyperintensities in the pons on T2-weighted images, atrophy of the cerebellum and middle cerebellar peduncles for MSA-C, and putaminal atrophy and hyperintensities in the dorsolateral aspects of putamen on T2-weighted images for MSA-P) (Savoiardo et al. 1990; Konagaya et al. 1994). Genetic testing for variants of alpha-synuclein had not been available for these patients.

Ultimately, 36 PD patients (19 males and 17 females) and 34 MSA patients (17 males and 17 females) were selected for this study. There were 15 patients with MSA-P (seven males and eight females) and 19 patients with MSA-C (10 males and nine females). The age at onset and the disease duration were examined in all patients. The severity of their Parkinsonism was estimated using the Hoehn and Yahr scale (HY) (Hoehn and Yahr 1967; Goetz et al. 2004). Daily doses of anti-Parkinson drugs were converted to Levodopa equivalents using an algorithm for estimating the parenteral doses of drugs for Parkinson's disease (Brennan and Genever 2010). The present retrospective study received the approval of the institutional review board (IRB) of National Center of Neurology and Psychiatry with a waiver of the need for patients' informed consent.

Controls were recruited from the community through local magazine advertisements and our website announcement. After the study was explained to each participant, written informed consent was obtained for participation. The collection of MR data for the controls was also approved by the institutional review board. The age-matched controls consisted of 64 participants (27 men and 37 women, mean age 65.1 ± 9.0 and 67.4 ± 5.9 years, respectively). For our evaluation of the age-dependent thermic changes, we also included young and middle-aged adult controls past their 20s. The complete adult control group consisted of 114 participants (47 men and 67 women, ages 28–89 and 29–78 years, mean ages 57.8 ± 14.6 and 55.9 ± 12.3 years, respectively).

### MR imaging

All patients and controls underwent MR imaging using a 3T system (Siemens Medical System, Erlangen, Germany). The protocol for neurodegenerative diseases including PD and MSA was axial T1-weighted images, axial T2-weighted images, coronal fluid attenuation inversion recovery (FLAIR) images, 3D-T1 weighted images (3DT1WI), and DWI. DWI was performed in the axial plane (repetition time (TR)/echo time (TE), 10,000/75 msec; matrix, 76 × 76; field of view (FOV), 23 × 23 cm; slice thickness 3 mm with no interslice gap; 55 continuous transverse slices, number of excitations (NEX), 1). Diffusion was measured along 12 noncollinear directions using a diffusion-weighted factor b in each direction of 1000 sec/mm(Geser et al. 2006), and one image was acquired without using any diffusion gradient.

T1-weighted images (TR/TE, 600/9.4 msec; matrix, 186 × 256; FOV, 22 × 22 cm; section thickness, 3 mm with gap of 1.2 mm; 35 continuous transverse slices; NEX, 1), T2-weighted images (TR/TE, 5000/81 msec; matrix, 348 × 512; FOV, 22 × 22 cm; section thickness, 3 mm with gap of 1.2 mm; 35 continuous transverse slices; NEX, 2), FLAIR images (TR/TE, 12000/94 msec; matrix 179 × 320; FOV, 22 × 22 cm; section thickness, 3 mm with gap of 0.9 mm; 42 continuous coronal slices; NEX, 1) and 3DT1WI (TR/TE, 1800/2.26 msec; matrix, 288 × 320; FOV 25 × 25 cm; effective section thickness, 0.8 mm with no interslice gap, 224 continuous sagittal slices; NEX, 1) were obtained.

### Temperature estimation

The diffusion coefficient of nonrestricted water molecules can be reliably measured using MRI. Several studies by Mills and Nagy et al. have shown that the CSF temperature can be estimated using this relationship. Mills showed the relationship between pure water diffusion and diffusivity, and Nagy et al. showed the ability of DWI to measure the CSF temperature based on the study by Mills (1973); Nagy et al. (2009).

We calculated the diffusion constant using the following equation:

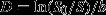
1where *D* is the diffusion constant (mm^2^/sec), *b* is the applied diffusion weighting (sec/mm^2^), and *S*_0_ and *S* are the voxel signal intensities of the reference and DWIs, respectively.

The *D* value was converted to the corresponding temperature using equation 2010:

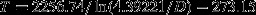
2where *T* is the temperature (°C). This DWI-based MR thermometry of the ventricles was calculated using an automated method developed by Sakai et al. (2012). The temperature estimates were only considered within the lateral ventricles, since this method is only applicable to nonrestricted water. According to a phantom study, the DWI-based temperatures show systematically higher values (1.2°C, almost constantly) than those measured by a thermometer and MRS-based temperatures (Sakai and Nakai 2013).

### Volumetric analyses of lateral ventricles

We performed volumetric segmentation of 3D T1-weighted brain MRI using FreeSurfer software (version 5.3.0, A. Martinos Center for Biomedical Imaging, Boston, MA), and the volume of bilateral lateral ventricles was estimated in all patients and controls.

### Statistical analyses

As there are significant differences in somatic thermoregulation between men and women, we analyzed the male and female patients/controls independently. The differences in age at onset, disease duration, and dose of anti-Parkinson drugs were evaluated using a two-sample t-test between PD and MSA patients. The differences in T_v_ values among the PD, MSA, and control groups were evaluated using an analysis of covariance (ANCOVA) controlling for age, and the volume of the lateral ventricles was analyzed in the same way. The *post hoc* test was carried out using Bonferroni's correction for multiple comparisons. The differences of T_v_ values were also evaluated using a two-sample t-test between MSA-P and MSA-C patients in the men and women, respectively. We assessed the correlation between age and T_v_ in the 114 adults of the all-age control group using the Pearson product-moment correlation coefficient, controlling for ventricular volume. We also assessed the correlation between ventricular volume and T_v_ in the all-age control group using the Pearson product-moment correlation coefficient. The statistical analyses were performed using SPSS Statistics for Windows 21.0 software (SPSS Japan, Tokyo).

## Results

**Figure 1:**
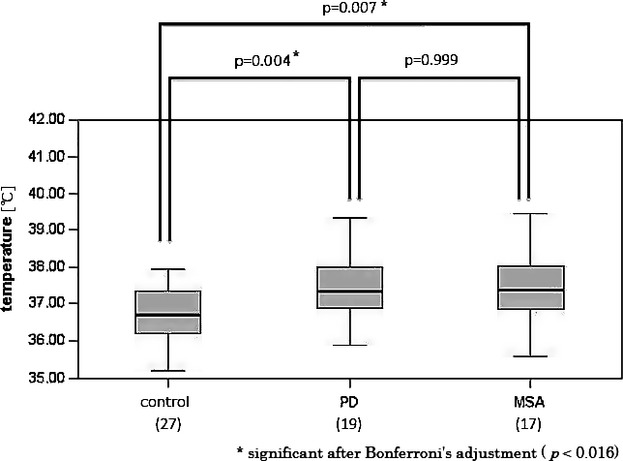
Measured ventricular CSF temperatures in male patients with PD (*n* = 19) and male patients with MSA (*n* = 17) compared to the normal male controls (*n* = 27). The ventricular CSF temperatures of the men with PD or MSA were significantly higher than those of the controls (*P* = 0.004 and *P* = 0.007, respectively).

The demographic and clinical characteristics of the participants are shown in Table 1. There were no significant differences in the age upon examination or the age of onset. The male PD patients had significantly longer disease durations (*P *=* *0.04) and took significantly more anti-Parkinson medication (*P *=* *0.05) than the male MSA patients. The breakdown of the Hoehn and Yahr scale scores varied in both the PD and MSA groups. The average T_v_ values of the male PD patients and the male MSA patients were 37.4 ± 0.8°C and 37.4 ± 1.0°C, respectively, and that of the male controls was 36.7 ± 0.7°C (Fig.1).

**Table 1:** Demographic and clinical characteristics of the healthy control subjects, PD patients, and MSA patients

Variable	Male				Female			
	Control	PD	MSA	*P*	Control	PD	MSA	*P*
	Mean ± SD	Mean ± SD	Mean ± SD		Mean ± SD	Mean ± SD	Mean ± SD	
No. of subjects	27	19	17		37	17	17	
Age	65.1 ± 9.0	68.1 ± 10.6	61.9 ± 9.3	0.17	67.4 ± 5.9	69.4 ± 4.4	65.5 ± 6.9	0.159
Onset age		64.9 ± 10.9	58.9 ± 9.7	0.16		65.5 ± 5.1	61.5 ± 6.6	0.056
Disease duration (months)		46.5 ± 25.1	31.5 ± 16.3	0.04		41.5 ± 26.6	44.5 ± 21.6	0.726
L-Dopa equivalent dose (mg)		283.9 ± 253.7	107.0 ± 258	0.05		274 ± 107.9	260 ± 289	0.854
Hoehn and Yahr scale		1.9 ± 0.8	1.4 ± 1.6	0.22		2.3 ± 0.8	1.8 ± 1.7	0.311

PD, Parkinson's disease; MSA, multiple system atrophy; SD, standard deviation.

**Figure 2:**
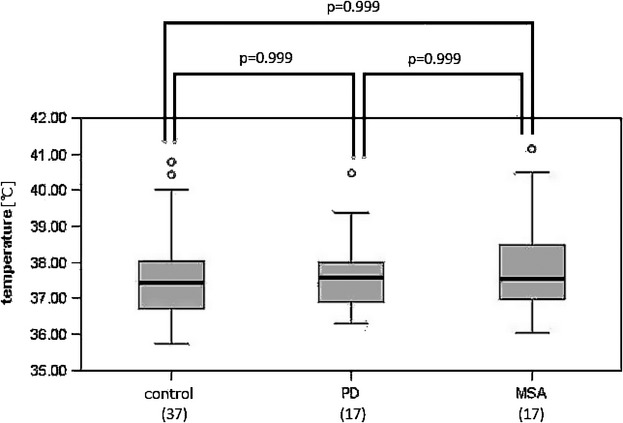
Measured ventricular CSF temperatures in female patients with PD (*n* = 17) or MSA (*n* = 17) compared to the normal female controls (*n* = 37). There were no significant differences between the PD or MSA patients and the controls.

The average T_v_ of the female PD patients and the female MSA patients were 37.6 ± 1.0°C and 37.9 ± 1.5°C, respectively, whereas that of the female controls was 37.5 ± 1.3°C (Fig.2). The T_v_ values of the male patients with PD or MSA were significantly higher than those of the male controls. This propensity was not recognized for women.

There were no significant differences between the T_v_ values of the MSA-P and MSA-C patients in the men or the women (men, *P* = 0.81; women, *P* = 0.63). The average volumes of lateral ventricles of the male PD and MSA patients were 36.7 ± 13.3 mL and 33.4 ± 14.3 mL, respectively, whereas that of the male controls was 32.4 ± 13.2 mL. There was no significant difference in the volumes of the bilateral ventricles among the PD, MSA, and normal groups in the men (*P* = 0.63). The average volumes of the bilateral ventricles of the female patients with PD and MSA were 30.7 ± 13.1 mL and 30.6 ± 18.3 mL, respectively, and that of the female controls was 24.0 ± 13.2 mL. The ventricular volumes of the female MSA patients were significantly larger than those of the female controls (*P* = 0.018). The ventricular volumes of the female PD patients were not significantly different from those of the female MSA patients or the female controls (*P* = 0.45 and *P* = 0.83, respectively).

**Figure 3:**
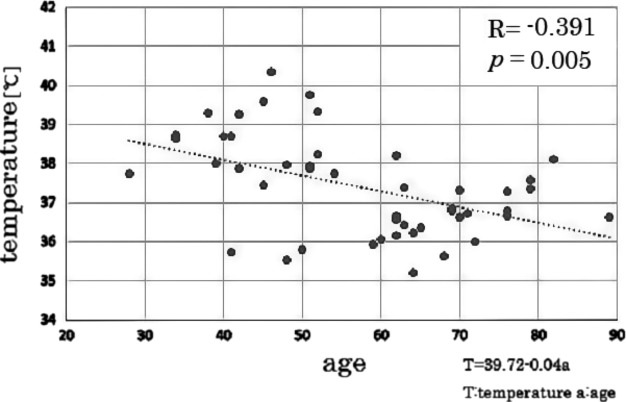
Correlation between age and ventricular CSF temperature in male controls. The dotted line indicates the result of linear fitting. Ventricular CSF temperature tends to decrease by age with a significant correlation controlling for ventricular volume.

**Figure 4:**
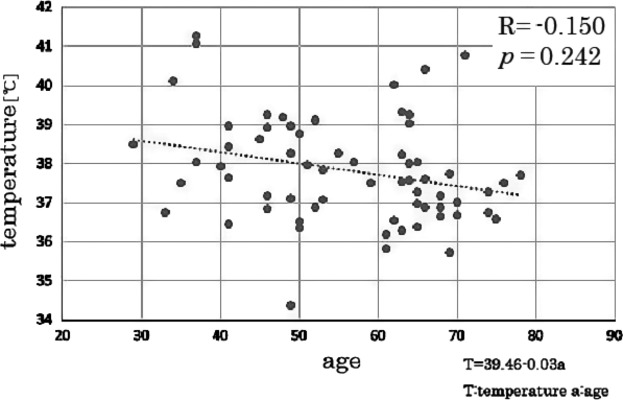
Correlation between age and ventricular CSF temperature in female controls. The dotted line indicates the result of linear fitting. There was no correlation between age and ventricular CSF temperature in female controls controlling for ventricular volume.

Our analysis revealed a significant negative correlation between ventricular size and T_v_ in the group of all-age male controls (correlation coefficient = −0.392, *P* = 0.007), but not in the all-age female controls (correlation coefficient = −0.169, *P* = 0.181). The partial correlation analysis revealed a significant negative correlation between age and T_v_ in the all-age male controls, controlling for the ventricular volume (Fig.3, partial correlation coefficient = −0.391, *P* = 0.005). This tendency did not occur in the all-age female controls (Fig.4, partial correlation coefficient = −0.150, *P* = 0.242).

## Discussion

The results of this study showed that by using DWI thermometry, significantly increased T_v_ values were revealed in males with PD or MSA compared to controls. Our analysis of a large number of healthy subjects with a wide range of ages (from 28 to 89) also revealed an age-dependent decline in T_v_, and that this decline was significant for men but not for women. The finding of a decline is in contrast to the PD and MSA male patients' high T_v_ values.

Our results indicated that disorders of cerebral thermic homeostasis occur in PD and MSA, which are both characterized by autonomic dysfunction.

As far as we know, there is only one study in which the cerebral temperatures of PD patients were calculated. In that study (Rango et al. 2012), MRS thermometry at the visual cortex was used; thus, their analysis was more accurate. However, they dealt with only 10 patients and did not independently assess men and women. There has been no report examining brain temperatures in MSA patients. Here, we assessed the T_v_ of PD and MSA patients for the first time using DWI thermometry, which is easier to use in a clinical setting than MRS. In addition, this study is the first to demonstrate changes in T_v_ associated with senility in men and women independently.

One previous report examined 45 healthy volunteers (between 19 and56 years of age) and found a correlation between age and T_v_ (Sakai et al. 2011). In this study, a larger number of controls (114 volunteers) with a wider age range (29–89 years old) were examined, and we assessed the men and women independently. Consequently, new findings such as gender differences and a continuous decline of T_v_ in the elderly men were demonstrated.

Brain parenchymal temperatures had been measured in previous studies using MRS. The temperature calculation is based on the measurement of the difference between the water resonance frequency, which is linearly temperature-dependent, and the N-acetyl aspartate methyl resonance frequency, which remains stable at different temperatures (Childs et al. 2007). However, MRS is a time-consuming and labor-intensive method. In contrast, DWI thermometry is based on a phenomenon in which an excited proton reduces echo signals by the free movement of water molecules. The higher the temperature is, the more the signal intensity is decreased due to more activated water molecules. T_v_ calculated by DWI depends simply on the voxel signal intensity, and it is influenced by various CSF conditions of lateral ventricles.

MRS thermometry measures the temperature of brain parenchyma and it is thus thought to be more accurate than DWI thermometry. However, to our knowledge there has been no in vivo study published that measured T_v_ using MRS. In phantom studies measuring artificial CSF, the temperature measured by DWI thermometry showed a good correlation with that measured simultaneously by MRS thermometry (Sakai and Nakai 2013).

DWI is easy to conduct and is one of the routine sequences in clinical MR examinations. A direct relationship between temperature and self-diffusion in water was demonstrated in a phantom study by Mills (1973). Kozak et al. (2010)confirmed this relationship on a water phantom using a 3.0-T clinical MR scanner, and they applied it for measurements of the temperature of the CSF within the lateral ventricles. With appropriate thresholds, temperature measurement can be successfully estimated by calculating the average number of voxels. The histogram curve-fitting methods were adopted in our custom-developed software, as discussed in previous studies (Sakai et al. 2012). DWI thermometry has been applied clinically to head injury (Tazoe et al. 2014), Moyamoya disease (Yamada et al. 2010), schizophrenia (Ota et al. 2014), multiple sclerosis (Sai et al. 2014), and in this study, for the first time, to Parkinsonian syndromes.

The temperature of the brain is dependent on cerebral metabolism, cerebral blood flow (CBF) and, to some degree, core body temperature. Heat production in the brain is closely related to the cerebral metabolic rates of glucose (CMRGlu) and oxygen (CMRO_2_), whereas CBF is essential to cool down the brain (Siesjo 1978). The age-dependent T_v_ decline determined in this study by DWI thermometry is probably due to decreases in CMRGlu and CMRO_2_ with age (Sakai et al. 2011). We detected a continuous decline of T_v_ in senescence in men, especially in the subjects over 60. Human studies examining acute ischemic stroke and Moyamoya disease have also shown significantly increased T_v_ compared to healthy subjects, suggesting that vascular impairment leads to a decline of CBF and eventually to elevated brain temperature and T_v_ (Karaszewski et al. 2006; Yamada et al. 2010). However, decreased T_v_ values have been reported in minor head trauma, reflecting a transient drop in cerebral metabolism (Mills 1973).

Mitochondrial function is the most important factor in cerebral metabolism. Indeed, Rango et al. (2014) showed that the brains of patients with mitochondrial diseases are hypothermic. Mitochondrial disease is caused by mitochondrial dysfunction, but it has also been reported in PD (Schapira 1994; Henchcliffe et al. 2008; Hattingen et al. 2009; Rango et al. 2012). The mitochondrial disorder of PD patients seems to act in a different way from that of classical mitochondriopathies. A patient with classical mitochondriopathy has a defect in mitochondrial oxidative phosphorylation due to reduced activity of the mitochondrial electron transport (Rango et al. 2014). Therefore, both oxygen and glucose consumption are decreased. These consumptions are increased in PD patients (Powers et al. 2008). The cause of this is still unknown, but an uncoupling of ATP production from oxidation in the mitochondria was reported in PD patients (Powers et al. 2008), and we speculate that wasteful consumption would result in an increased demand for oxygen and glucose in PD patients. These mechanical differences may bring opposite results.

We also observed the rise of T_v_ in MSA patients, but no change in the mitochondrial respiratory chain function of MSA patients had been noted previously (Gu et al. 1997). One study of goldfish demonstrated decreased body temperature when dopamine was injected into the heat center, that is, the anterior aspect of the nucleus preopticus periventricularis (Wollmuth et al. 1989).

Aside from mitochondrial disorders, the failure of preganglionic dopaminergic pathways could contribute to thermic alterations in PD and MSA pathology. Medication including L-DOPA should also be considered. Leenders et al. (1985) demonstrated that L-DOPA caused an increase in regional CBF, which could decrease brain temperature in PD patients. In the pars compacta of the substantia nigra, the transient receptor potential cation channel, subfamily V, members 3/4 (TRPV3/4) are activated in expression systems by high temperature (Guatteo et al. 2005). The direct relationships between dopaminergic pathways and the decline of cerebral temperature remain to be investigated, but it is almost certain that the dopaminergic system itself is also under the influence of cerebral temperature.

As our measurement of T_v_ was indirect, the possibility of the influence of elevated body temperature in PD or MSA patients cannot be eliminated. However, this possible influence was examined and denied by the results of a previous study: Rango et al. examined body and brain temperatures very precisely, keeping their PD patients and normal controls at the constant temperature at 22°C for 1 h before examination, and the results indicated no difference in body temperature between the PD patients and controls (Rango et al. 2012). The body core temperature in MSA patients has not been reported, to the best of our knowledge. As our volumetric analysis revealed no significant difference in ventricular volume between the male PD/MSA patients and normal controls, the change in T_v_ in both patient groups cannot be explained by the effect of ventricular size. Although pathologic brain atrophy is correlated with lower CSF temperature, the T_v_ values of the male PD/MSA patients were elevated, which is a paradoxical result.

The effect of aging on the body core temperature is not yet known. Some studies showed decreased body temperature in the elderly (Howell 1948; Roghmann et al. 2001; Güneş and Zaybak 2008), but no studies have reported increased body core temperature with aging. Marion et al. (1991) demonstrated that exclusion of the following factors negated any relationship between chronological age and body core temperature in a sample of 93 volunteers aged 62–96 years: diabetes and neurological disorders, low body weight and consumption of less than two meals per day, smoking, lack of self-sufficiency, alcohol intake >3 oz. per week, and the use of various medications. However, complete exclusion of these pathologic factors is difficult even in those who are assumed to be “healthy.” It would be better in practice to assume that older age leads to lower body temperature.

Brain atrophy and ventricular enlargement with aging should be also considered here, since it is expected that T_v_ declines when the heat produced spreads to more of the CSF. A previous study showed no correlation between T_v_ and ventricular volume in normal controls, probably because of their narrow range of age (Sakai et al. 2011). In this study's population, there was a greater variation in ventricular size than in the previous study, and a negative correlation between T_v_ and ventricular volume was demonstrated for the men. However, a significant negative correlation between age and T_v_ in the all-age male controls was also observed, controlling for the ventricular volume. Age still seems to be an important factor that influences T_v_. In any case, further prospective examinations are needed to clarify the relationship between T_v_ and brain atrophy.

In the healthy female controls of this study, the correlation between age and T_v_ was not detected as it was in men. Menstrual cycle has a great effect on the core body temperature, and it can also alter CSF temperature. Estrogen cools the body temperature before ovulation, and progesterone warms the body temperature after ovulation and until menstruation. The secretion of these hormones does not stop completely after menopause; they gradually decrease (Buxton and Atkinson 1948). The effect of aging on T_v_ may be obscured in the presence of varied phases of the menstrual cycle in each individual case.

Although the menstrual cycle influences body temperature, it cannot explain this phenomenon in postmenopausal women. One study showed that a higher core body temperature was correlated with higher levels of luteinizing hormone in older postmenopausal women (Murphy and Campbell 2007), suggesting that sex hormones and gonadotropins still affect thermal controls in the elderly. Brooks et al. (1997) reported that prolonged estrogen replacement therapy acts centrally to decrease core body temperature. Thus, the individual variations in hormonal levels and body temperature may be large enough to mask the significant difference in T_v_ values between patients and controls.

In summary, this study showed a significant rise in the T_v_ of male patients with PD or MSA compared to that of healthy male controls using DWI thermometry. A negative correlation between age and T_v_ in the male controls was shown, controlling for the ventricular volume. An automated temperature calculation method for DWI thermometry would provide more information with which to determine patients' metabolic status.

Although further research is needed on the clinical use of DWI thermometry (as there are great variations in T_v_ among individuals), DWI images are easier to obtain than MRS images, and the measurement of temperature can be achieved along with daily clinical scans in a short time, and even retrospectively. Our results demonstrated variations of T_v_ among individuals of different ages and gender. The altered T_v_ in men with PD and MSA indicates the possible involvement of mitochondrial and autonomic dysfunction. We believe that DWI thermometry has the potential to elucidate the pathophysiology of Parkinsonian syndromes.

## Study Limitations

A limitation of our study is that the body temperatures of the patients were not always available, as this was a retrospective study. Although the brain temperature may be partially dependent on the core body temperature, a prior study of normal subjects using DWI thermometry depicted no correlation between T_v_ and the body temperature itself (Sakai et al. 2011). Some studies demonstrated a difference in the nocturnal core body temperature between PD and MSA patients, and decreased nocturnal core body temperatures were observed in PD, but not in MSA (Pierangeli et al. 2001). The difference of body temperature in PD or MSA patients in light of gender is not discussed in the existing literature. Further investigations comparing the ventricular and core body temperatures may explain the difference between PD/MSA patients and normal controls. Another potential limitation of this study is that various pathological features of the CSF suggest that we cannot expect the calculated temperature to be as accurate as in laboratory experiments using pure water. The pulsatile movement of the CSF increases the diffusion coefficient. In certain locations, such as the foramen of Monro, overestimation of the temperature could be problematic. Conversely, the substrates contained in the CSF, including glucose, protein, electrolytes, and blood cells, lead to underestimation of the brain temperature. The possibility cannot be excluded that an altered composition of CSF with aging leads to an “apparent” thermic change in ventricular CSF. This may also be the case for PD and MSA patients. More research involving the DWI and MRS method are needed to obtain more accurate CSF temperature estimates.

## Conflict of Interest

None declared.
